# An adjuvanted zoster vaccine elicits potent cellular immune responses in mice without QS21

**DOI:** 10.1038/s41541-022-00467-z

**Published:** 2022-04-22

**Authors:** Hyo Jung Nam, Sung Jun Hong, Ara Lee, Jiyeon Kim, Sangho Lee, Corey Casper, Darrick Carter, Steven G. Reed, George Simeon, Eui-Cheol Shin

**Affiliations:** 1 Laboratory of Immunology and Infectious Diseases, Graduate School of Medical Science and Engineering, Korea Advanced Institute of Science and Technology, Daejeon, Republic of Korea; 2 MOGAM Institute for Biomedical Research, Gyeonggi-do, Republic of Korea; 3 GC Pharma, Gyeonggi-do, Republic of Korea; 4 Infectious Disease Research Institute, Seattle, WA USA; 5 PAI Life Sciences Inc., Seattle, WA USA; 6 CUREVO Vaccine, Seattle, WA USA; 7 Present Address: HDT bio, Seattle, WA USA

**Keywords:** Recombinant vaccine, Adjuvants

## Abstract

Herpes zoster (HZ) is caused by reactivation of latent varicella-zoster virus (VZV) when VZV-specific cellular immunity is insufficient to control reactivation. Currently, Shingrix, which contains the VZV gE protein and GSK’s AS01_B_ adjuvant composed of liposomes formulated with cholesterol, monophosphoryl lipid A (MPL) and QS21, is used for prevention of HZ. However, reactogenicity to Shingrix is common leading to poor patient compliance in receiving one or both shots. Here, we evaluated the immunogenicity of a newly formulated gE protein-based HZ vaccine containing Second-generation Lipid Adjuvant (SLA), a synthetic TLR4 ligand, formulated in an oil-in-water emulsion (SLA-SE) without QS21 (gE/SLA-SE). In VZV-primed mouse models, gE/SLA-SE-induced gE-specific humoral and cellular immune responses at comparable levels to those elicited by Shingrix in young mice, as both gE/SLA-SE and Shingrix induce polyfunctional CD4^+^ T-cell responses. In aged mice, gE/SLA-SE elicited more robust gE-specific T-cell responses than Shingrix. Furthermore, gE/SLA-SE-induced T-cell responses were sustained until 5 months after immunization. Thus, QS21-free, gE/SLA-SE is a promising candidate for development of gE-based HZ vaccines with high immunogenicity—particularly when targeting an older population.

## Introduction

Varicella zoster virus (VZV) is a neurotropic human alpha herpesvirus that causes varicella (also known as chickenpox) and herpes zoster (HZ; also known as shingles)^1^. Varicella, caused by primary infection with VZV, is a self-limited disease in healthy children but may be accompanied by serious complications—including bacterial sepsis, pneumonia, and encephalitis in immunocompromised individuals^2^. After resolution of varicella, VZV establishes latency in neurons of the cranial nerve, dorsal root, and autonomic ganglia along the entire neuroaxis. HZ is caused by reactivation of latent VZV and characterized by a painful blistering rash^3–5^. Patients with HZ may suffer from sequelae including a constant, severe stabbing or burning pain known as post-herpetic neuralgia (PHN)^6^.

VZV-specific cellular immunity controls reactivation of VZV among latently infected individuals. In aged or immunocompromised individuals, VZV cannot be controlled due to insufficient cellular immunity, resulting in HZ^7^. Aging that is characterized by a decline in cellular immunity is associated with not only an increased incidence of HZ, but also increased risk of PHN^8^. In addition, HZ is more severe and lasts longer in immunocompromised patients and they are at high risk of developing serious complications and disseminated disease^9^.

Current strategies for preventing HZ are vaccination of the elderly using a live attenuated virus vaccine, Zostavax (MSD, USA), or an adjuvanted subunit vaccine, Shingrix (GSK, Belgium). Zostavax is administered subcutaneously in a single dose and reduces the burden of illness by 61.1% and the incidence of PHN by 66% among people ≥60 years of age^10^. However, the efficacy of preventing HZ decreases with increasing age, with 41% vaccine efficacy among people aged 70–79 years, which is further decreased down to 18% in individuals >80 years old^11^. Shingrix is an adjuvanted subunit vaccine containing VZV gE protein and AS01_B_ adjuvant. Shingrix is administered intramuscularly in two doses and has a vaccine efficacy of 97.2% in adults ≥50 years of age. Furthermore, Shingrix shows good vaccine efficacy in adults ≥70 years of age and an efficacy of 97.9% for the prevention of HZ. In addition, Shingrix induces durable responses, resulting in 87.9% efficacy in the fourth year after vaccination^12,13^. Shingrix has a preferred recommendation from the Advisory Committee on Immunization Practices (ACIP) for the prevention of HZ and related complications for immunocompetent adults ≥50 years of age and immunocompetent adults who previously received Zostavax^14^.

Although Shingrix has remarkable efficacy (>90%) in preventing HZ, the immunization is accompanied by high reactogenicity with frequent solicited reports of injection site pain and systemic reactions within 7 days after vaccination. Furthermore, Shingrix-immunized subjects are reported to have increased severe (grade 3) symptoms, both systemic (3–4.8-fold higher in Shingrix group) and at the injection site (23.8–42.5-fold higher in Shingrix group) compared to placebo subjects in both the ZOE-50 and ZOE-70 trials^12,13^. The reactogenicity induced by Shingrix could be explained by the strong immunostimulatory effect of the AS01_B_ adjuvant, which is composed of liposomes formulated with cholesterol, monophosphoryl lipid A (MPL), and QS21^15^.

MPL is a detoxified derivative of the lipopolysaccharide from *Salmonella minnesota* that stimulates innate immune responses through TLR4 signaling^16^. The adjuvant system AS04—consisting of an aluminum salt and MPL—has been licensed for use in preventive vaccines for hepatitis B virus and human papillomavirus. MPL directly stimulates innate immune cells via TLR4 signaling and consequently enhances antigen-specific adaptive immune responses—including humoral and cellular responses^17^.

QS21 is a saponin purified from the bark extract of *Quillaja saponaria* Molina (fraction 21), and QS21 is thought to mediate its adjuvant activity by accumulating CD169^+^ resident macrophages in the draining lymph node and by activating caspase-1^18–20^. Although QS21 has strong activity for stimulating helper-1 T (T_H1_) and cytotoxic T lymphocyte (CTL) responses, the high reactogenicity of saponins like QS-21 are a downside and include injection site pain and undesirable hemolytic effects^21^. High reactogenicity of QS21 has been reported in previous clinical studies. In an HIV-1 vaccine trial, QS21-adjuvanted HIV-1 antigens had moderate or severe local pain/tenderness, categorized as a severe burning sensation, in two-thirds of individuals^22,23^. In an influenza vaccine trial, injection site pain and myalgia were significantly more frequent in the QS21-adjuvanted inactivated influenza vaccine group compared to the group without QS21^24^.

Adjuvant technology has advanced since the development of AS01, and novel adjuvants, formulations, and component materials have been developed. New synthetic TLR4 agonists have also been developed since MPL. Glucopyranosyl Lipid Adjuvant (GLA) is a TLR4 agonist based on the structure of natural TLR4 ligands. GLA has a potent adjuvant activity for enhanced T_H1_ responses, as well as IgG2c-skewed humoral responses in mice. The safety and immunogenicity of GLA formulated in an oil-in-water emulsion (GLA-SE) has been assessed in humans. The overall safety profile of GLA-SE with vaccine antigen is acceptable, and adverse events associated with vaccines are mild-to-moderate and transient^25–27^. Second-generation Lipid Adjuvant (SLA) is a next-generation, synthetic TLR4 ligand optimized for binding to the human MD2 component of the MD2/TLR4 innate signaling complex^28^. The adjuvant effect of SLA formulated in an oil-in-water emulsion (SLA-SE) was tested in mice and humans as a mixture with LEISH-F3, a leishmaniasis vaccine. LEISH-F3 adjuvanted with SLA-SE elicited strong CD4^+^ T-cell responses in both mice and humans^29^.

In the present study, we evaluated the immunogenicity of a newly formulated gE protein-based, QS21-free HZ vaccine in murine models. This vaccine contains recombinant VZV gE protein and SLA-SE without QS21. We demonstrate that the SLA-SE-adjuvanted gE (gE/SLA-SE) vaccine robustly induces both humoral and cellular immune responses. Durable maintenance of vaccine-induced cellular immunity is also shown. In addition, we examined vaccine-induced immune responses in aged mice and found that our gE/SLA-SE vaccine elicits stronger immune responses than the lead competitor, Shingrix. Our current results suggest that the gE/SLA-SE vaccine has potential to be developed as a novel QS21-free HZ vaccine.

## Results

### Immunization using gE with SLA-SE significantly enhances antigen-specific immune responses

**Fig. 1:**
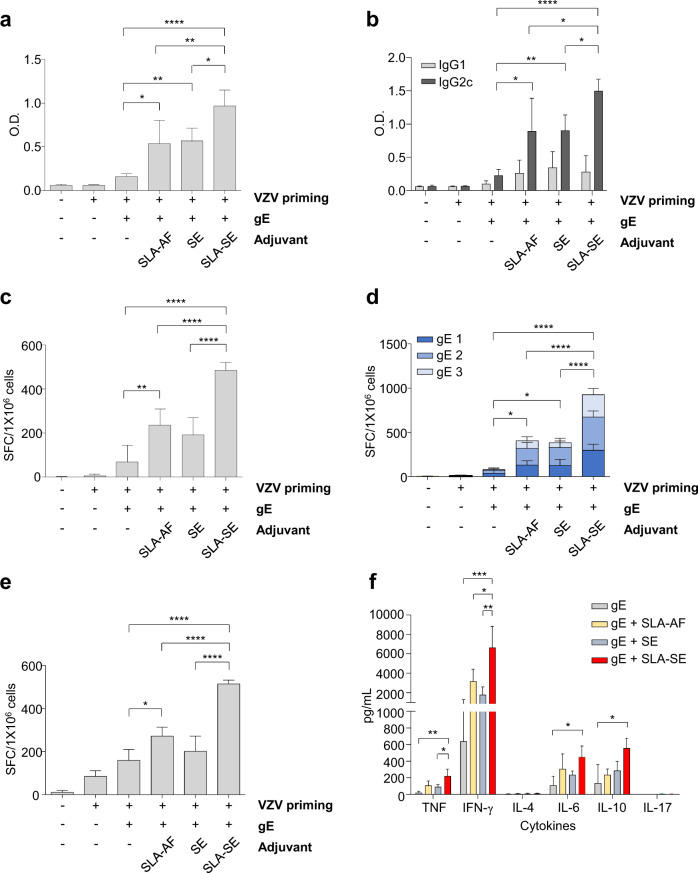
gE- and VZV-specific immune responses induced by gE/SLA-AF, gE/SE, or gE/SLA-SE. C57BL/6 mice were primed with live attenuated VZV, and then immunized once with gE (5 µg), gE (5 µg)/SLA in aqueous formulation (SLA-AF, 5 µg of SLA), gE (5 µg)/oil-in-water emulsion (SE, 2% oil), or gE (5 µg)/SLA-SE (5 µg SLA in 2% oil) 4 weeks later. Two weeks after immunization, serum was obtained from immunized mice to examine humoral immunity and spleen cells prepared to examine cell-mediated immunity. **a** gE-specific total IgG antibody response by IgG ELISA using 100,000-fold diluted serum samples from immunized mice. **b** gE-specific IgG1 and IgG2c antibody response by IgG ELISA using 100,000-fold diluted serum from immunized mice. **c**–**e** Antigen-specific, IFN-γ-producing T-cell populations were detected by mouse IFN-γ ELISPOT assay using spleen cells from immunized mice upon antigenic stimulation with **c** gE protein, **d** three pools of 62 gE OLPs (15-mer, 5-mer overlap): gE 1 (20 peptides), gE 2 (20 peptides), and gE 3 (22 peptides) or **e** VZV-infected cell lysate. **f** Measurement of cytokine secretion upon re-stimulation with gE OLP by cytometric bead array assay. Data are presented as means and standard deviations. *P < 0.05, **P < 0.01, ***P < 0.005, ****P < 0.001 (ordinary one-way ANOVA). *n* = 4 mice per group.

In the present study, we examined the immunogenicity of recombinant gE protein in combination with SLA-based adjuvants in mice. We primed mice by administering live-attenuated VZV four weeks before immunization. We examined the effects of different adjuvant formulations [SLA in an aqueous formulation (“SLA-AF”), an oil-in-water emulsion alone without a TLR4 agonist (“SE”), and SLA-SE] by evaluating gE-specific antibody and T-cell responses 2 weeks after a single immunization. We evaluated gE-specific serum IgG responses using an enzyme-linked immunosorbent assay (ELISA) and gE-specific T-cell responses by IFN-γ ELISpot assays following ex vivo stimulation of splenocytes with gE protein. Among the three different adjuvants, SLA-SE had the strongest adjuvant effect on gE-specific total IgG and IgG2c, but not IgG1 (Fig. 1a and b). IgG2c-dominant humoral responses indicate T_H1_-skewed immune responses^30^. We also evaluated gE-specific T-cell responses in IFN-γ ELISpot assays following ex vivo stimulation of splenocytes with either gE protein or overlapping peptides (OLPs) spanning the gE protein and found that SLA-SE most significantly enhanced the gE-specific IFN-γ-producing T-cell responses (Fig. 1c, d). Similar results were obtained when splenocytes were stimulated ex vivo with VZV-infected cell lysate (Fig. 1e). We examined gE-specific production of various cytokines in culture supernatants from splenocytes stimulated with gE OLPs and found that SLA-SE most significantly increased gE-specific IFN-γ secretion among the three different adjuvants and resulted in the production of TNF, IL-6, and IL-10, but not of IL-4 and IL-17 (Fig. 1f). Thus, gE/SLA-SE immunization biasis T-cell responses toward T_H1_ rather than T helper-2 (T_H2_) or T helper-17 (T_H17_) responses.

### gE/SLA-SE vaccine elicits gE-specific immune responses comparable to those elicited by Shingrix in young mice

**Fig. 2:**
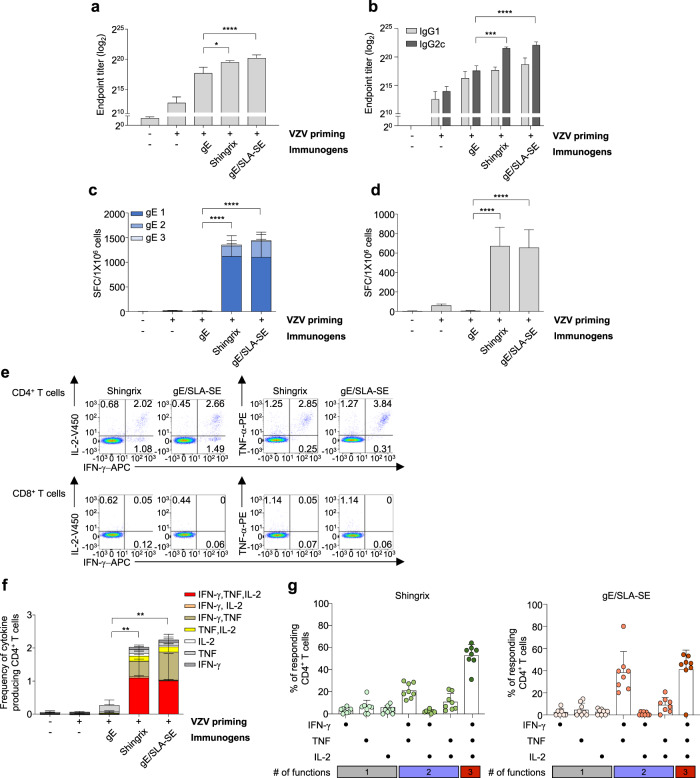
Comparison of gE- or VZV-specific immune responses of gE/SLA-SE with Shingrix in young mice. Six-week-old C57BL/6 mice were primed with live attenuated VZV, and then immunized twice with gE (5 µg), gE (5 µg)/SLA-SE (10 µg SLA in 2% oil), or Shingrix (1/10 of human dose, gE [5 µg]/AS01_B_ [5 µg MPL and 5 µg QS21]) at a 4-week interval. Four weeks after the second immunization, serum was obtained from immunized mice to examine humoral immunity and spleen cells prepared to examine cell-mediated immunity. **a** gE-specific total IgG endpoint antibody titer (log_2_ transformed) using serum from immunized mice. **b** gE-specific IgG1 and IgG2c antibody titer (log_2_ transformed) using serum from immunized mice. **c**, **d** IFN-γ-producing T-cell populations by mouse IFN-γ ELISPOT assay using spleen cells from immunized mice upon antigenic stimulation with (**c**) three pools of 62 gE OLPs (15-mer, 5-mer overlap): gE 1 (20 peptides), gE 2 (20 peptides), and gE 3 (22 peptides) or (**d**) VZV-infected cell lysate. **e** Representative FACS plots of cytokine-secreting CD4^+^ and CD8^+^ T-cell populations in Shingrix or gE/SLA-SE immunized mice upon gE OLP stimulation. **f** Measurement of the frequency of cytokine-producing CD4^+^ T cells for IFN-γ, TNF, and IL-2 upon gE OLP stimulation. **g** CD4^+^ T-cell polyfunctionality analyzed by every possible combination of functions using Boolean analyses in spleen cells from either Shingrix immunized mice or gE/SLA-SE immunized mice upon gE OLP stimulation. Data are representative of two independent experiments, and data are presented as means and standard deviations. *P < 0.05, **P < 0.01, ****P < 0.001 (ordinary one-way ANOVA). *n* = 4 (control), 8 mice per group.

On the basis of the results presented in Fig. 1, we chose SLA-SE as an adjuvant for gE immunization. Next, we compared the gE-specific immune responses elicited by gE/SLA-SE with those elicited by Shingrix. Six-week-old mice were primed with live attenuated VZV, twice immunized with gE, Shingrix, or gE/SLA-SE, and sacrificed 4 weeks after the second immunization to evaluate the gE-specific immune responses. Immunization with gE/SLA-SE elicited gE-specific total IgG, IgG1, and IgG2c responses as evaluated by serum ELISA (Fig. 2a and b) and T-cell responses as evaluated by IFN-γ ELISpot assays following ex vivo stimulation of splenocytes with gE OLPs (Fig. 2c) or VZV-infected cell lysate (Fig. 2d). Responses were similar (not statistically significant) to those elicited by Shingrix with a trend towards higher responses in the gE/SLA-SE groups. We also evaluated gE-specific T-cell responses by performing intracellular cytokine staining (ICS) following ex vivo stimulation of splenocytes with gE OLPs. When CD4^+^ and CD8^+^ T cells were analyzed separately, we found that CD4^+^ T cells were a major source of various cytokines, including IL-2, IFN-γ, and TNF, whereas CD8^+^ T cells did not produce cytokines at a similar level as expected (Fig. 2e). In the analysis of CD4^+^ T cells by ICS, gE/SLA-SE elicited similar levels of gE-specific cytokines as Shingrix (Fig. 2f). In particular, both gE/SLA-SE and Shingrix-generated gE-specific polyfunctional CD4^+^ T cells that produced multiple cytokines simultaneously (Fig. 2g). Taken together, the results show that gE/SLA-SE elicits gE-specific immune responses comparable to those elicited by Shingrix.

### gE/SLA-SE elicits stronger T-cell responses than Shingrix in aged mice

**Fig. 3:**
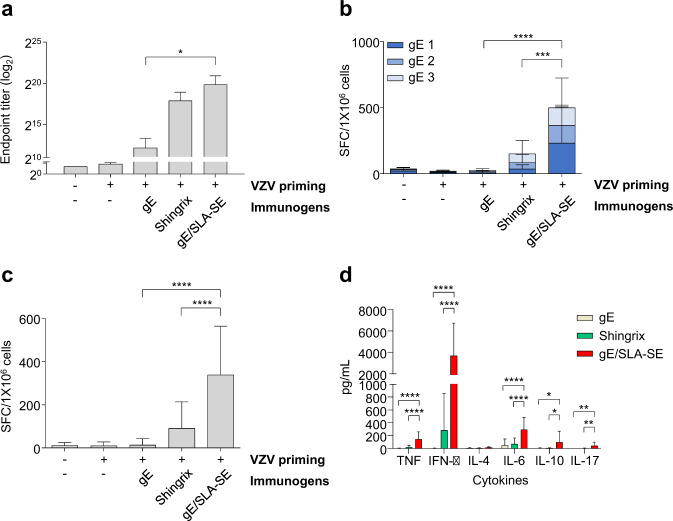
gE- or VZV-specific immune responses induced by gE/SLA-SE or Shingrix in elderly mice. Eighteen-month-old mice were primed with live attenuated VZV and immunized twice with gE (5 µg), gE (5 µg)/SLA-SE (10 µg SLA in 2% oil), or Shingrix (1/10 human dose, gE [5 µg]/AS01_B_ [5 µg MPL and 5 µg QS21]) at a 4-week interval. Four weeks after the second immunization, serum was obtained from immunized mice to examine humoral immunity and spleen cells prepared to examine antigen-specific cell-mediated immunity. **a** gE-specific total IgG endpoint antibody titer (log_2_ transformed) using serum from immunized mice. **b**, **c** Detection of antigen-specific IFN-γ-secreting T-cell numbers by mouse IFN-γ ELISPOT assay using spleen cells from immunized mice upon ex vivo stimulation with (**b**) three pools of 62 gE OLPs (15-mer, 5-mer overlap): gE 1 (20 peptides), gE 2 (20 peptides), and gE 3 (22 peptides) or (**c**) VZV-infected cell lysate. **d** Measurement of cytokine secretion upon stimulation with gE OLP by cytometric bead array assay. Data are pooled from two independent experiments and presented as individual data points with means and standard deviations. *P < 0.05, **P < 0.01, ***P < 0.005, ****P < 0.001 (ordinary one-way ANOVA). *n* = 4-6 (control), 8–10 mice per group.

Next, we evaluated vaccine-induced immune responses in aged mice. Eighteen-month-old mice were primed with live attenuated VZV, twice immunized with gE, Shingrix, or gE/SLA-SE, and sacrificed 4 weeks after the second immunization. gE/SLA-SE induced significantly higher gE-specific IgG ELISA titers than gE alone, but Shingrix did not (Fig. 3a). Importantly, gE/SLA-SE elicited significantly stronger gE-specific T-cell responses than Shingrix in IFN-γ ELISpot assays following ex vivo stimulation of splenocytes with gE OLPs (Fig. 3b) or VZV-infected cell lysate (Fig. 3c). We also examined gE-specific production of various cytokines in culture supernatants from splenocytes ex vivo stimulated with gE OLPs and found that gE/SLA-SE elicits significantly increased gE-specific secretion of IFN-γ, TNF, IL-6, IL-10, and IL-17 compared to gE or Shingrix (Fig. 3d). These findings demonstrate that gE/SLA-SE exhibits stronger T-cell immunogenicity than Shingrix in aged mice.

### Long-term maintenance of gE/SLA-SE-induced T-cell responses

**Fig. 4:**
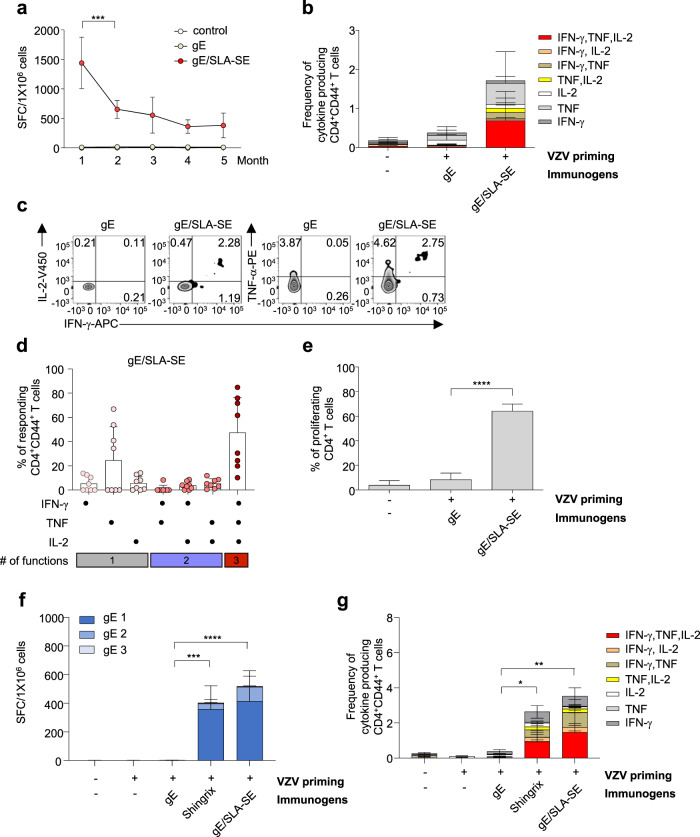
The durability and functionality of gE-specific T-cell responses by immunization of gE/SLA-SE. Six-week-old mice were primed and immunized twice with either gE (5 µg) or gE (5 µg)/SLA-SE (10 µg SLA in 2% oil) at a 4-week interval. **a** Detection of gE-specific IFN-γ-secreting T-cell numbers by mouse IFN-γ ELISPOT assay upon stimulation with three pools of 62 gE OLPs (15-mer, 5-mer overlap): gE 1 (20 peptides), gE 2 (20 peptides), and gE 3 (22 peptides). Mice (*n* = 4 (control), 8 (group) per each month) were sacrificed each month from 1 to 5 months after the second immunization to examine the durability of the antigen-specific T-cell responses. **b** Measurement of the frequency of cytokine-secreting T cells among CD4^+^ CD44^hi^ T cells by intracellular cytokine assay upon gE OLP stimulation 5 months after the second immunization. **c** Representative FACS plots of IFN-γ, TNF, and/or IL-2-secreting CD4^+^CD44^+^ T-cell populations 5 months after the second immunization in gE or gE/SLA-SE immunized mice. **d** CD4^+^ T-cell polyfunctionality analyzed by every possible combination of functions using Boolean analysis in spleen cells from gE/SLA-SE immunized mice 5 months after the second immunization upon gE OLP stimulation. **e** The percentage of proliferating gE-specific CD4^+^ T cells using Violet Proliferation Dye 450 in spleen cells from either gE or gE/SLA-SE immunized mice 5 months after the second immunization upon 6-day stimulation with gE OLP. **f**, **g** Comparison of the durability and functionality of gE-specific T-cell responses induced by gE/SLA-SE or Shingrix. Six-week-old mice were primed and immunized twice with gE (5 µg), gE (5 µg)/SLA-SE (10 µg SLA in 2% oil), or Shingrix (1/10 human dose, gE [5 µg]/AS01_B_ [5 µg MPL and 5 µg QS21]). Three months after the second immunization, spleen cells were prepared to examine cell-mediated immunity. **f** Detection of gE-specific IFN-γ-secreting T-cell numbers by mouse IFN-γ ELISPOT assay using spleen cells from immunized mice upon ex vivo stimulation with three pools of 62 gE OLPs (15-mer, 5-mer overlap): gE 1 (20 peptides), gE 2 (20 peptides), and gE 3 (22 peptides). **g** CD4^+^ T-cell polyfunctionality analyzed by every possible combination of functions using Boolean analysis in spleen cells from immunized mice upon ex vivo gE OLP stimulation. Data are representative of two independent experiments, and data are presented as means and standard deviations. ***P < 0.005, ****P < 0.001 (ordinary one-way ANOVA).

Finally, we evaluated the longevity of the vaccine-induced T-cell responses in mice immunized with gE or gE/SLA-SE. Six-week-old mice were primed with live attenuated VZV, twice immunized with gE or gE/SLA-SE and maintained for up to 5 months for evaluation of the durability of gE-specific T-cell responses. When gE-specific T-cell responses were evaluated by IFN-γ ELISpot assays following ex vivo stimulation of splenocytes with gE OLPs, we found that gE/SLA-SE-induced T-cell responses peaked at 1 month but significantly decreased by 2 months after the second immunization. Thereafter, gE-specific T-cell responses were well maintained up to 5 months. In contrast, significant gE alone-induced T-cell responses were not observed (Fig. 4a). We also examined the polyfunctionality of vaccine-induced T-cell responses 5 months after the second immunization by ICS following ex vivo stimulation of splenocytes with gE OLPs. We found that the CD4^+^ T cells of gE/SLA-SE-immunized mice produced multiple cytokines 5 months after immunization (Fig. 4b, c). We also found that gE/SLA-SE-immunized mice maintained a high degree of polyfunctionality of gE-specific CD4^+^ T cells up to 5 months after the second immunization (Fig. 4d). Similar findings were observed in CD4^+^ T-cell proliferation assays following ex vivo stimulation of splenocytes with gE OLPs (Fig. 4e). Taken together, these results indicate that gE/SLA-SE successfully elicits long-term sustained T-cell memory against gE. We also performed a similar analysis with gE, Shingrix, or gE/SLA-SE-immunized mice 3 months after the second immunization. In IFN-γ ELISpot assays, both the Shingrix and gE/SLA-SE groups exhibited significantly higher IFN-γ-producing T-cell responses than the gE group (Fig. 4f). However, there was no difference between the Shingrix and gE/SLA-SE groups. Similar results were observed for ICS of CD4^+^ T cells for multiple cytokines (Fig. 4g).

## Discussion

Here we report the immunogenicity of recombinant gE protein with SLA-SE, a synthetic TLR-4 agonist formulated in an oil-in-water emulsion, in VZV-primed C57BL/6 mice. Consistent with previous reports, recombinant gE antigen without any adjuvant was poorly immunogenic in regards to both humoral and cellular immune responses^31^. These data show that vaccination of VZV-primed mice with gE/SLA-SE induced strong humoral responses to gE antigen and increased the frequency of polyfunctional CD4^+^ T cells, which was durable for several months. Notably, gE/SLA-SE elicited significant enhancement of gE-specific T-cell responses when compared to the commercial benchmark, Shingrix, in aged mice.

Currently, there are two vaccines available for the prevention of HZ. After approval by the U.S. Food and Drug Administration (FDA) in May 2006, Zostavax was licensed for the prevention of HZ in more than 55 countries, with more than 34 million doses distributed worldwide^32^. Zostavax exhibits 51% overall vaccine efficacy and 67% efficacy against PHN^33^. Shingrix was approved by the U.S. FDA in October 2017 and has 91% overall vaccine efficacy and 91% efficacy against PHN; more than 3 million doses of this vaccine has been given^33,34^. Comparative studies for the vaccine-mediated immune responses elicited by Zostavax or Shingrix have shown that Shingrix can generate more than 10-times more memory T cells than Zostavax^35^. By including a strong adjuvant, AS01_B_, Shingrix elicits strong antigen-specific immune responses and durable protection against the onset of HZ. However, it is also responsible for increasing the frequency of both local and systemic side effects to vaccination, resulting in a rate of grade 3 severe reactions of 10% in both the ZOE-50 and ZOE-70 trials^12,13,36^. Therefore, it would be worthwhile to develop vaccines for HZ prevention that have lower reactogenicity.

Among the various types of vaccines for the prevention of infectious diseases, subunit vaccines, which contain a recombinant protein from the pathogen, have been well-studied. However, the major limitation of subunit vaccines is limited immunogenicity, particularly low T-cell immunogenicity^37^. Adjuvants are important in enhancing and/or shaping antigen-specific immune responses, and there have been numerous attempts to develop vaccine adjuvants^38^. Ligands for pattern-recognition receptors (PRRs) have been well-studied as vaccine adjuvants due to the activation of innate immune responses, and TLR4 ligands—such as MPL—are the most well-known adjuvant components in regards to both safety and immunogenicity^39^. SLA was developed to specifically target the human TLR4 receptor and has been shown to induce a TRIF pathway-biased shift, which increases type I interferon production and influences T_H1_ differentiation^29,40^. In addition, the immunostimulatory effect of the ligands for PRRs can be further enhanced when the molecules are combined with appropriate adjuvant formulations, such as aluminum salts, liposomes, and oil-in-water emulsions^41,42^. The adjuvant combination should be chosen based on the type of immune response desired, antigen characteristics, and age of the target population^43^. In the present study, we examined the adjuvanticity of SLA-SE as an HZ vaccine candidate in a mouse model. SLA-SE significantly enhanced the gE-specific antibody and T-cell responses compared to SLA-AF or SE in young mice, and the immune response was comparable to the response elicited by the leading licensed HZ vaccine, Shingrix.

It is well known that immune function declines with age due to a decrease in the number of naïve cells and increase in dysfunctional memory cells, known as immune senescence. These immunological changes in the elderly are clinically important, resulting in an increased opportunity for new infections and reactivation of latent viruses^44^. Immune senescence is one of the reasons why most approved vaccines exhibit lower vaccine immunogenicity and efficacy in the elderly compared to younger adults^45^. As the target population for adjuvanted subunit HZ vaccines is older people, we tried to evaluate vaccine immunogenicity in aged mice. Mice are a preferred animal model for immunological studies and aged mice exhibit age-acquired immunological defects, characterized by the deterioration of antibody responses—especially in germinal center function and somatic hypermutation—and by dysfunction of antigen-specific T-cell responses in naïve and memory T cells^46^. When we compared the humoral immune responses after immunization with gE/SLA-SE between young mice and aged mice, there was no significant difference in the gE-specific IgG responses (Figs. 2a and 3a). Although the overall antigen-specific T-cell responses after immunization with gE/SLA-SE were lower in aged mice than young mice, gE/SLA-SE elicited strong T_H1_-skewed immune responses, indicating that gE/SLA-SE could be effective even in aged populations.

In summary, we demonstrated that the gE/SLA-SE vaccine is highly immunogenic in regards to generating VZV-specific antibodies and CD4^+^ T-cells with durable responses, and can induce strong T-cell responses in elderly mice. These observations suggest that the vaccine candidate could be appropriate for further development as a new prophylactic vaccine against HZ.

## Methods

### Preparation of recombinant VZV gE protein

The coding sequence of VZV gE (strain Dumas), including the extracellular domain but not the transmembrane and anchor domains, was cloned into the pMSID2-gE vector. The recombinant VZV gE protein was expressed in Chinese hamster ovary (CHO) DG44 cells and the secreted product purified from culture supernatant by a series of column chromatographies and including viral inactivation, concentration, and diafiltration steps. The purified gE protein was analyzed by SDS–PAGE and size-exclusion chromatography (Supplementary Fig. 1) and the purity was over 95%. The protein solution was sterilized by filtration and stored at −70 °C until use.

### Adjuvant

The synthetic TLR4 agonist SLA was synthesized by Corden Pharma Switzerland and formulated either as an aqueous suspension (SLA-AF) or a stable oil-in-water emulsion (SLA-SE) by IDRI (Seattle, WA)^29^. SE was formulated by IDRI^47^.

### Animals and immunization

Mouse husbandry and all the procedures involving mice were performed with approval from the Institutional Animal Care and Use Committee of GC Pharma and MOGAM Institute of Biomedical Research (approval number: GC-17-004). C57BL/6 female mice (5–6 weeks old) were purchased from Orient Bio and maintained under specific pathogen-free conditions. For the aged mouse experiments, 8-month-old C57BL/6 female mice were purchased from Samtako and aged up to 18 months. To mimic natural VZV infection^31^, mice were primed by subcutaneous administration of the live attenuated Varicella vaccine (GC Pharma). Four weeks after priming, mice were immunized intramuscularly in the tibialis muscle once (Fig. 1) or twice, 4 weeks apart (Figs. 2–4) with the different vaccine formulations (*n* = 4–10/each group) or phosphate-buffered saline (PBS) as a control. For Shingrix, lyophilized gE antigen was reconstituted with AS01_B_ adjuvant according to the manufacturer’s instructions, and 1/10 human dose (in a volume of 50 µL) was dissolved in PBS before immunization, yielding a formulation containing 5 µg gE and adjuvant with 5 µg MPL and 5 µg QS21. For gE/SLA-SE, 5 µg gE and either 5 µg SLA in 2% SE (Fig. 1) or 10 µg SLA in 2% SE (Figs. 2–4) were dissolved in PBS before immunization. Two (Fig. 1) or 4 weeks (Figs. 2–4) after the last immunization, blood samples were taken from the immunized mice to obtain serum, and the mice were sacrificed to obtain the spleen.

### Anti-gE-specific IgG ELISA

An ELISA was performed to detect anti-gE-specific IgG antibodies in serum from immunized mice using gE protein as the coating antigen. Briefly, gE protein was diluted in PBS and added to a 96-well ELISA plate for protein coating at a final concentration of 1 µg/mL. After overnight incubation at 4 °C, plates were washed twice with PBS containing 0.05% TWEEN 20 (PBST) and saturated with 2% bovine serum albumin (BSA, Sigma) in PBS for 1 h at room temperature. After washing four times with PBST, diluted serum samples were added to the plate and incubated for 2 h at room temperature, and then washed four times with PBST. The plates were incubated with horseradish peroxidase-conjugated anti-mouse total IgG, IgG1 or IgG2c (SouthernBiotech) and diluted in PBS containing 2% BSA for 1 h at room temperature. After washing four times with PBST, visualization was achieved using tetramethylbenzidine substrate solution (TMB, Seracare).

### Mouse IFN-γ ELISPOT assays

The number of cells secreting IFN-γ was determined by IFN-γ ELISPOT assays^48^. Briefly, splenocytes were prepared from immunized mice and ex vivo stimulated with either immunized protein or customized gE OLP pools (Peptron) for 20 h. Total 62 OLPs (15-mer, 5-mer overlap) were aliquoted into 3 groups, gE 1 (20 peptides), gE 2 (20 peptides), and gE 3 (22 peptides) and used for stimulation. Culture media or 0.1% DMSO (Sigma-Aldrich) was used as a negative control. For the VZV-specific T-cell responses, VZV-infected cell lysates (Microbix Biosystems) were used for ex vivo antigen stimulation, and MRC-5 mock cell lysates (Microbix Biosystems) were used as a negative control^49^. Samples were incubated for 20–24 h. IFN-γ-secreting cells were detected using a mouse IFN-γ ELISPOT kit (BD Bioscience) and plates were read using an ELISpot reader (AID GmbH). The number of antigen-specific IFN-γ-secreting cells were calculated by subtracting the spot number in the negative control from the spot number from antigen stimulation with protein, OLP, or lysate.

### Intracellular cytokine staining assays

The frequency of antigen-specific CD4^+^ T cells secreting IFN-γ, TNF, and/or IL-2 was detected using flow cytometry^31^. Briefly, splenocytes were ex vivo stimulated with either the gE OLP pool of total 62 peptides or 0.3% DMSO as a formulation control in the presence of anti-CD28 and anti-CD49d costimulatory antibodies (BD Biosciences), and then incubated for 2 h at 37 °C. Following the antigen stimulation, Brefeldin A (BD Biosciences) was added to splenocytes and further incubated overnight. After stimulation, splenocytes were stained with a mixture of 7-AAD, anti-CD3-FITC, anti-CD8-PE-Cy7, and anti-CD4-V500 (BD Biosciences). For ICS, surface-labeled splenocytes were permeabilized with Cytofix/Cytoperm buffer (BD Biosciences) and labeled with a mixture of anti-TNF-α-PE, anti-IL-2-V450, and anti-IFN-γ-APC (BD Biosciences). Stained samples were analyzed using an LSRII flow cytometer (BD Biosciences) and FlowJo software (FlowJo, LLC).

### Quantitation of secreted cytokines upon antigen stimulation

To detect the levels of cytokines secreted after antigen stimulation, splenocytes were ex vivo stimulated with customized gE OLP pools (15-mer, 5-mer overlap) or 0.3% DMSO as a formulation control for 3 days at 37 °C. After antigen stimulation, supernatants were harvested by centrifugation and secreted cytokine concentrations determined using a mouse Th1/Th2/Th17 CBA kit (BD Biosciences) according to the manufacturer’s instructions. Samples were analyzed using an LSRII flow cytometer (BD Biosciences) and FCAP Array software (BD Biosciences).

### T-cell proliferation assays

Antigen-specific T-cell proliferation was measured using Violet Proliferation Dye 450 (VPD450; BD Biosciences) upon antigen stimulation. Splenocytes were washed twice with PBS and labeled with VPD450 at a final concentration of 1 µM. VPD450-labeled splenocytes were incubated with customized gE OLP pools (15-mer, 5-mer overlap) for 6 days at 37 °C, and then stained with a mixture of anti-CD3-FITC, anti-CD8-PE, 7-AAD, and anti-CD4-APC (BD Biosciences). Cells with increasingly lower VPD450 mean fluorescence intensities were considered to be proliferated cells upon antigen stimulation. Stained samples were analyzed using an LSRII flow cytometer (BD Biosciences) and FlowJo software (FlowJo, LLC).

### Statistical analysis

All data are expressed as means and standard deviations (SD). Statistical analyses were performed using Prism software 9.0 (GraphPad Software, Inc.). The differences among groups were determined using ordinary one-way ANOVA, assuming normal distribution. Differences were considered significant when *p* < 0.05.

### Reporting summary

Further information on research design is available in the Nature Research Reporting Summary linked to this article.

## Supplementary information

Supplementary Information

REPORTING SUMMARY

## Data Availability

The data that support the findings of this study are available from the corresponding author upon reasonable request.
