# Supplementary material for: An adjuvanted zoster vaccine elicits potent cellular immune responses in mice without QS21
Source: NPJ Vaccines. 2022 Apr 22;7:45. doi: 10.1038/s41541-022-00467-z (PMC9033770; doi:10.1038/s41541-022-00467-z)
Supplement: Supplementary file 1 — Supplementary Information [file 41541_2022_467_MOESM1_ESM.pdf]

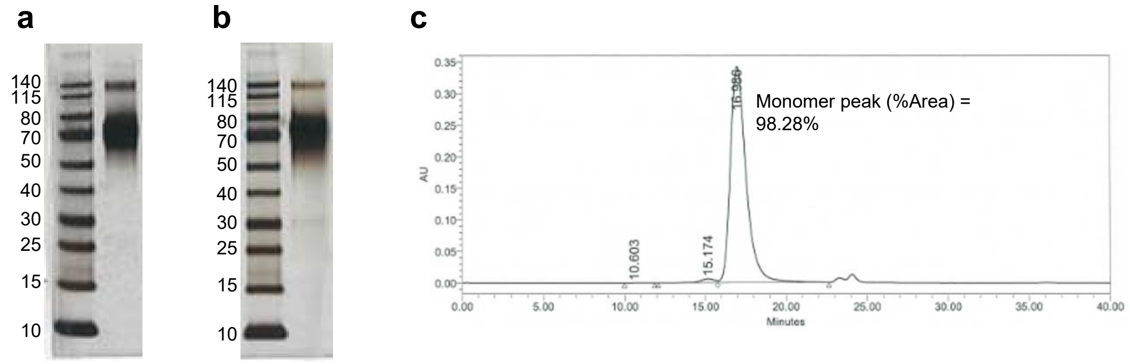

Supplementary Figure 1. SDS-PAGE and chromatography results for purified gE protein. (a, b) The purity of recombinant VZV gE protein was verified using SDS-PAGE followed by silver staining. SDS-PAGE was performed under (a) non-reducing and (b) reducing conditions. (c) The purity of the recombinant VZV gE protein was verified by size exclusion chromatography using a BioSep-SEC-s3000 column.
