# Supplementary material for: An adjuvanted zoster vaccine elicits potent cellular immune responses in mice without QS21
Source: NPJ Vaccines. 2022 Apr 22;7:45. doi: 10.1038/s41541-022-00467-z (PMC9033770; doi:10.1038/s41541-022-00467-z)
Supplement: Supplementary file 2 — REPORTING SUMMARY [file 41541_2022_467_MOESM2_ESM.pdf]

nature portfolio

Corresponding author(s): Professor Eui-Cheol Shin

Last updated by author(s): Jan 1, 2021

Reporting Summary

Nature Portfolio wishes to improve the reproducibility of the work that we publish. This form provides structure for consistency and transparency in reporting. For further information on Nature Portfolio policies, see our Editorial Policies and the Editorial Policy Checklist.

Statistics

For all statistical analyses, confirm that the following items are present in the figure legend, table legend, main text, or Methods section.

n/a	Confirmed	
☐	☒	The exact sample size (*n*) for each experimental group/condition, given as a discrete number and unit of measurement
☐	☒	A statement on whether measurements were taken from distinct samples or whether the same sample was measured repeatedly
☐	☒	The statistical test(s) used AND whether they are one- or two-sided<br>*Only common tests should be described solely by name; describe more complex techniques in the Methods section.*
☒	☐	A description of all covariates tested
☐	☒	A description of any assumptions or corrections, such as tests of normality and adjustment for multiple comparisons
☐	☒	A full description of the statistical parameters including central tendency (e.g. means) or other basic estimates (e.g. regression coefficient) AND variation (e.g. standard deviation) or associated estimates of uncertainty (e.g. confidence intervals)
☐	☒	For null hypothesis testing, the test statistic (e.g. *F*, *t*, *r*) with confidence intervals, effect sizes, degrees of freedom and *P* value noted<br>*Give P values as exact values whenever suitable.*
☒	☐	For Bayesian analysis, information on the choice of priors and Markov chain Monte Carlo settings
☒	☐	For hierarchical and complex designs, identification of the appropriate level for tests and full reporting of outcomes
☒	☐	Estimates of effect sizes (e.g. Cohen's *d*, Pearson's *r*), indicating how they were calculated

Our web collection on statistics for biologists contains articles on many of the points above.

Software and code

Policy information about availability of computer code

Data collection	No software was used for data collection.
Data analysis	Statistical analyses were performed using Prism software 9.0

For manuscripts utilizing custom algorithms or software that are central to the research but not yet described in published literature, software must be made available to editors and reviewers. We strongly encourage code deposition in a community repository (e.g. GitHub). See the Nature Portfolio guidelines for submitting code & software for further information.

Data

Policy information about availability of data

All manuscripts must include a data availability statement. This statement should provide the following information, where applicable:

- Accession codes, unique identifiers, or web links for publicly available datasets
- A description of any restrictions on data availability
- For clinical datasets or third party data, please ensure that the statement adheres to our policy

The data that support the findings of this study are available from the corresponding author upon reasonable request.

Field-specific reporting

Please select the one below that is the best fit for your research. If you are not sure, read the appropriate sections before making your selection.

☒ Life sciences ☐ Behavioural & social sciences ☐ Ecological, evolutionary & environmental sciences

For a reference copy of the document with all sections, see nature.com/documents/nr-reporting-summary-flat.pdf

Life sciences study design

All studies must disclose on these points even when the disclosure is negative.

Sample size	The sample size were chosen based on the previous experience that can be analyzed for the statistical significance.
Data exclusions	No data were excluded from the analyses.
Replication	The experiments were replicated, and all the attempts at replication were successful.
Randomization	In the experiment, we immunized naive mice to examine the antigen specific immunogenicity. There was no need to randomize the animals used for the experiments.
Blinding	For the study, we immunized mice and performed immunological assays to examine antigen specific immunogenicity induced by immunogens. Thus, blinding was not relevant to this study.

Reporting for specific materials, systems and methods

We require information from authors about some types of materials, experimental systems and methods used in many studies. Here, indicate whether each material, system or method listed is relevant to your study. If you are not sure if a list item applies to your research, read the appropriate section before selecting a response.

Materials & experimental systems

n/a	Involved in the study	
☐	☒	Antibodies
☐	☒	Eukaryotic cell lines
☒	☐	Palaeontology and archaeology
☐	☒	Animals and other organisms
☒	☐	Human research participants
☒	☐	Clinical data
☒	☐	Dual use research of concern

Methods

n/a	Involved in the study	
☒	☐	ChIP-seq
☐	☒	Flow cytometry
☒	☐	MRI-based neuroimaging

Antibodies

Antibodies used

- goat anti-mouse IgG(H+L)-HRP : Southern Biotech. 1031-05.
- goat anti-mouse IgG1-HRP : Southern Biotech. 1070-05.
- goat anti-mouse IgG2c-HRP : Southern Biotech. 1079-05.
- mouse IFN-g ELISPOT pair : BD. 551881.
- anti-mouse CD3-FITC : BD. 553062.
- anti-mouse CD8-PE.Cy7 : BD. 552877.
- anti-mouse CD4-V500 : BD. 560782.
- anti-mouse TNFa-PE : BD. 554419.
- anti-mouse IL-2-V450 : BD. 560546.
- anti-mouse IFN-g-APC : BD. 554413.
- anti-mouse CD8-PE : BD. 553032.
- anti-mouse CD4-APC : BD. 553051.

Validation

All antibodies in this study were validated by manufacturers.

Eukaryotic cell lines

Policy information about cell lines

Cell line source(s)	Chinese hamster (CHO-DG44 cell line)
Authentication	Cell line was authenticated by DNA fingerprinting.

Mycoplasma contamination	The cell line was negative for mycoplasma contamination test.
Commonly misidentified lines (See ICLAC register)	There is no misidentified cell line in this study.

Animals and other organisms

Policy information about studies involving animals; ARRIVE guidelines recommended for reporting animal research

Laboratory animals	C57BL/6, female
Wild animals	The study did not involve wild animals.
Field-collected samples	The study did not involve samples collected from the field.
Ethics oversight	Mouse husbandry and all the procedures involving mice were performed with approval from the Institutional Animal Care and Use Committee of GC Pharma and MOGAM Institute of Biomedical Research (approval number: GC-17-004).

Note that full information on the approval of the study protocol must also be provided in the manuscript.

Flow Cytometry

Plots

Confirm that:

☒ The axis labels state the marker and fluorochrome used (e.g. CD4-FITC).

☒ The axis scales are clearly visible. Include numbers along axes only for bottom left plot of group (a 'group' is an analysis of identical markers).

☒ All plots are contour plots with outliers or pseudocolor plots.

☒ A numerical value for number of cells or percentage (with statistics) is provided.

Methodology

Sample preparation	Splenocytes were prepared from immunized mice.
Instrument	LSRII flow cytometer (BD Biosciences) was used for the Flow Cytometry.
Software	FlowJo software was used for the analysis.
Cell population abundance	In the experiment, we did not perform cell sorting.
Gating strategy	First, Lymphocytes were gated by FSC/SSC, and then Single Cells were gated by FSC-A, H, W within the lymphocyte population. Among the Single Cells, Live cells were gated by 7-AAD negative population, and then CD3 positive cells were gated within the live cells. Either CD4 positive or CD8 positive cells were gated within the CD3 positive cells, and then cytokine positive cells were further gated within either CD4 or CD8 positive cells.

☒ Tick this box to confirm that a figure exemplifying the gating strategy is provided in the Supplementary Information.
